# Replacement of Soybean Meal with Heat-Treated Canola Meal in Finishing Diets of Meatmaster Lambs: Physiological and Meat Quality Responses

**DOI:** 10.3390/ani10101735

**Published:** 2020-09-24

**Authors:** Mpolokeng Sekali, Victor Mlambo, Upenyu Marume, Manny Mathuthu

**Affiliations:** 1 Department of Animal Science, Faculty of Natural and Agricultural Sciences, North-West University, P Bag x2046, Mmabatho 2735, South Africa; Msekali8@gmail.com (M.S.); Upenyu.Marume@nwu.ac.za (U.M.); 2 Food Security and Safety Niche area, Faculty of Natural and Agricultural Sciences, North-West University, P Bag x2046, Mmabatho 2735, South Africa; 3 School of Agricultural Sciences, Faculty of Agriculture and Natural Sciences, University of Mpumalanga, P Bag x11283, Mbombela 1200, South Africa; 4 Centre for Applied Radiation Science and Technology, Faculty of Natural and Agricultural Science, North-West University, P Bag X2046, Mmabatho 2735, South Africa; Manny.Mathuthu@nwu.ac.za

**Keywords:** dietary protein, growth performance, hematology, heat treatment, sheep

## Abstract

**Simple Summary:**

The use of soybeans as a dietary protein source in ruminant diets is environmentally, economically and socially unsustainable. It is for this reason that canola meal (CM) has emerged as a more sustainable alternative protein source to soybean meal (SBM). However, expeller CM is quickly broken down in the rumen resulting in inefficient utilization by rumen microbes, while a small rumen bypass protein fraction means that few of the essential amino acids in CM reach the small intestine. Consequently, the utility of CM protein as an alternative to soybean protein for high-producing ruminants is lower, requiring pre-feeding treatments such as heating to enhance its feed value. This study investigated whether heat treatment could improve the feed value of CM protein sufficiently to partially (50%) or completely substitute SBM in finishing diets of Meatmaster lambs. The results showed that while CM can completely replace SBM in diets of lambs without compromising growth performance and meat quality, heat treatment of CM did not offer any additional benefits.

**Abstract:**

The study investigated whether heat treatment (190 °C for 90 min) could improve the quality of expeller canola meal (CM) protein in finishing diets of Meatmaster lambs. Five isonitrogenous and isoenergetic diets were formulated by partially (50%) or completely replacing SBM as the major protein source with untreated or heat-treated CM in a commercial lamb finishing diet. Diets were randomly allocated to 40 lambs (24.73 ± 1.311 kg; 4.5 months old) for 77 days. No dietary differences were observed for growth performance, carcass characteristics and meat quality. Diets had no effect (*p* > 0.05) on blood parameters, except for hemoglobin and aspartate aminotransferase (AST). Lambs that were fed the control diet had higher hemoglobin concentration compared with lambs on CM-containing diets. The highest AST value was observed when SBM was completely replaced with untreated CM (159.88 IU/L), while complete replacement with heat-treated CM resulted in the lowest AST value (103.25 IU/L). All lambs had a body condition score of 3 at slaughter. It was concluded that heat treatment did not improve the protein value of CM. However, CM inclusion promoted similar growth performance and meat quality parameters in lambs as SBM. Untreated CM can be used to completely substitute SBM in finishing diets of Meatmaster lambs.

## 1. Introduction

Soybeans (*Glycine max*) have several uses that include direct consumption by humans, production of cooking oil, protein source in animal feed and biodiesel production. These multiple uses create a huge demand for limited supplies of this commodity resulting in high market prices. In addition, successful cultivation of soybeans in South Africa (SA) requires high levels of inputs such as fertilizers, pesticides, herbicides and irrigation water; as a result, the country imports about half a million tons of soybean annually [1]. The competition between humans and animals as well as the high input requirements for growing soybeans in SA contribute to the unsustainability of using soybean meal (SBM) as an animal feed ingredient. Indeed, the demand for soybeans in SA is largely driven by expanding animal production enterprises [2], where SBM is preferred as a concentrated source of dietary protein (44–48%) in livestock feeds. The high protein and energy content [3] as well as low fiber content of SBM are desirable attributes in the formulation of high-energy animal finishing diets. Locally produced soybeans can only meet a small fraction of the demand [4], hence the reliance on imports to bridge the gap between demand and supply. Therefore, there is a need for exploration of less expensive, locally produced, alternative dietary protein sources for livestock, especially those with limited direct feed value for humans.

A possible alternative dietary protein source is canola (*Brassica napus*) meal (CM), a by-product of oil extraction from canola seeds. About 1.5 million tons of canola grain is produced annually from 75–85,000 ha of land mostly in the Western Cape province of SA [5]. The increasing land area under canola cultivation and yields in SA make it a readily available potential protein supplement for livestock feeding [6]. Canola meal has a high protein (37%) content [7,8], which is a rich source of the sulfur-containing amino acids, methionine and cysteine [9]. However, unlike SBM, the usefulness of mechanically extracted CM as a protein supplement for ruminants is limited by high rumen degradability [10,11]. This reduces nitrogen utilization efficiency by rumen microbes as well as the rumen bypass protein fraction that is critical for high-producing ruminants. Rumen degradability of CM is high due to its high soluble-protein content [12]. Therefore, it is imperative that strategies to increase the rumen undegradable protein (RUP) fraction of CM are employed to improve its protein quality in finishing lamb diets. It has been shown that protecting dietary protein from rumen microbial degradation enables more absorbable amino acids to reach the small intestine, thus improving animal performance [13].

A variety of chemical and physical strategies to protect dietary proteins from extensive rumen microbial degradation have been investigated [14,15]. Physical treatments such as irradiation [16], autoclaving [17] and direct heating are preferred due to the ease of application as well as safety considerations. Heat treatment may lead to an alteration of protein structure [18] and facilitates Maillard reaction between reducing sugars and amino acids to form complexes that are more resistant to enzymatic hydrolysis than normal peptides, allowing for increased RUP and hence post-ruminal amino acid supply. Nevertheless, the nature of influence of heat-treated canola meal on the performance and meat quality characteristics in lambs is not known. Therefore, this study was designed to investigate whether heat treatment (190 °C for 90 min) can improve the quality of CM protein sufficiently enough to partially (50%) or completely substitute SBM in finishing diets without compromising the growth performance and meat quality of Meatmaster lambs. It was hypothesized that partial or complete replacement of SBM with heat-treated expeller CM as a dietary protein source in finishing diets of Meatmaster lambs would result in similar or improved growth performance and meat quality characteristics.

## 2. Materials and Methods

All animal handling practices and welfare considerations for the lambs followed the recommendations of the National Society for the Prevention of Cruelty to Animals (NSPCA) in South Africa. All experimental procedures were approved by the University Ethics Committee, North-West University, South Africa (Ethical Clearance No.: NWU-00523-16-A9).

### 2.1. Study Site

The feeding experiment was conducted at the North-West University Farm, Molelwane, South Africa (25°40.459′ S, 26°10.563′ E, altitude: 1400 m above sea level). The ambient temperature during the feeding trial ranged from 27 °C to 37 °C during summer and from −3 °C to 25 °C in winter. The annual rainfall in this area ranges between 300 and 600 mm.

### 2.2. Protein Sources and Experimental Diet Formulation

Soybean meal was purchased from Opti Feeds Pvt. Ltd., (Lichtenburg, South Africa). Canola meal, mechanically extracted in an expeller, was purchased from Southern Oil (Pty) Ltd. Company in Western Cape, South Africa. The CM was then toasted at a temperature of 190 °C for 90 min in a Memmert Universal Oven UF450plus (Lasec Pvt Ltd., Pretoria, South Africa). The heat treatment regimen was selected based on a preliminary assessment of the efficacy of temperature and heat duration on the in vitro ruminal degradability of CM. Five isonitrogenous and isocaloric dietary treatments (Table 1) were formulated (Opti Feeds Pvt. Ltd., Lichtenburg, South Africa) to meet the nutritional requirements of lambs [19] as follows: (1) commercial lamb finishing diet in which SBM was the major protein source (CON); (2) commercial finishing diet in which 50% of SBM was replaced by untreated canola meal (CM50); (3) commercial finishing diet in which all SBM was replaced by untreated canola meal (CM100); (4) commercial finishing diet in which 50% of SBM was replaced by heat-treated canola meal (HCM50); and (5) commercial finishing diet in which all SBM was replaced by heat-treated canola meal (HCM100).

### 2.3. Chemical Analyses

Canola meal, SBM and experimental diets were ground to pass through a 2-mm sieve using a Wiley Mill Model ED5 (Arthur H. Thomas Company, PA, USA) and subjected to chemical analyses. Dry matter (DM; method no. 930.15) [20] was determined by oven-drying the samples at 105 °C for 12 h and ash content (method no. 942.05) [20] was determined after incineration at 550 °C for 12 h. Nitrogen content was determined using the Kjeldahl method (method no. 976.05) [20] and crude protein (CP) was calculated as N × 6.25. Neutral detergent fiber (NDF) and acid detergent fiber (ADF) were determined according to Van Soest et al. [21] using an ANKOM 220 fiber analyzer (ANKOM Technology Corporation, NY, USA). Heat-stable bacterial α-amylase, but not sodium sulfite (Na_2_SO_3_), was used for NDF analysis. Acid detergent lignin (ADL) was determined by solubilizing cellulose with 72% sulfuric acid as described by Van Soest et al. [21]. The fiber fractions were then expressed in g/kg DM inclusive of residual ash. The chemical composition of experimental diets is presented in Table 2.

### 2.4. Animal Management

Forty Meatmaster lambs (24.73 ± 1.311 kg initial liveweight), aged 4.5 months old, were ear-tagged, vaccinated against pulpy kidney (1 mL/lamb) using Pulpyvax^®^ (Intervet South Africa (Pty) Ltd., Johannesburg, South Africa) and treated for internal parasites using Virbamax First Drench (6 mL/lamb) (Virbac RSA (Pty) Ltd., Centurion, South Africa). The lambs were randomly allotted to the five experimental diets and housed individually in pens (3.4 m^3^), each fitted with a drinker and a feeder. The lambs were then adapted to the experimental diets and pens for 10 days before measurements commenced. Lambs had free access to clean drinking water at all the times. The supply of experimental diets was restricted at 4% body weight per day with the daily ration being split for morning (7 h) and afternoon (14 h) feeding sessions. At this feeding rate, there were daily feed refusals that were collected and weighed every morning before the next feeding.

### 2.5. Growth Performance

The feeding trial lasted for 77 days. Feed offered was weighed and refusals were collected every morning before feeding. Feed intake (FI) was then calculated as the difference between the feed offered and the refusals. Each lamb was weighed weekly and the average daily gain (ADG) was calculated as:

(1) $$ADG\left(t_{0},T\right)=\frac{W\left(T\right)-W\left(t_{0}\right)}{T-t_{0}}$$

where $t_{0}$ is the initial time, T is the final time (77th day), W(T) is the final body weight and $W\left(t_{0}\right)$ is the initial body weight. Feed conversion ratio was calculated for the entire feeding period as the ratio of feed intake to weight gain.

### 2.6. Blood Collection and Analysis

On the last day of the trial, before morning feeding, blood samples were collected from the jugular vein by venipuncture using an 18-gauge needle into purple top, ethylenediaminetetraacetic acid-coated vacutainer tubes for hematological analysis using the IDEXX LaserCyte Hematology Analyzer (IDEXX Laboratories (Pty) Ltd., Johannesburg, South Africa). White blood cells, red blood cells (RBCs), hematocrit/packed cell volume (PCV), hemoglobin (Hgb), mean cell volume (MCV), mean cell hemoglobin (MCH), mean corpuscular hemoglobin concentration (MCHC), red cell distribution width (RDW) and platelets were determined. The differential count included neutrophil, lymphocyte, monocyte, eosinophil and basophil counts. Red top Vacuette^®^ Serum Clot Activator tubes (Greiner Bio-One, GmbH, Frickenhausen, Germany) were used to collect blood for serum biochemistry analyses. Clotted blood was centrifuged in a macrocentrifuge to generate serum for the determination of total protein (TP), creatinine, albumin (ALB) and aspartate transaminase (AST) using the automated IDEXX Vet Test Chemistry Analyzer (IDEXX Laboratories (Pty) Ltd., Johannesburg, South Africa).

### 2.7. Carcass Measurements

At the end of the feeding trial, lambs were fasted overnight with free access to water and transported to the abattoir for slaughter in the morning. The lambs were electrically stunned with a dual point electrode placed to the head, bled for about 2 min, skinned and eviscerated. The head (sectioned at the atlano-occipital joint) and feet (sectioned at the metacarpal and metatarsal joints) were removed. Hot carcass weight (HCW) was determined after removal of internal organs and perinephric–pelvic fat and the carcasses were then refrigerated at −4 °C for 24 h. Ultimate pH (pH_u_) and temperature were measured 24 h post-slaughter on the longissimus dorsi muscle (central area of the loin) using a Corning Model 4 pH–temperature meter (Corning Glass Works, Medfield, MA, USA). Cold carcass weight (CCW) and carcass length were measured at 24 h post-slaughter. The dressing-out percentage was determined as a proportion of HCW to slaughter weight. Carcasses were classified according to the official South African Carcass Classification System for age (by dentition) and fatness (visual appraisal) [22] and carcass conformation (CCONF) was also recorded. Carcass fatness was classified on a scale of 0–6 (0, no visual fat cover; 1, very lean; 2, lean; 3, medium; 4, fat; 5, over-fat; and 6, excessively over-fat) by cutting through the skin in the loin area and measuring the subcutaneous fat with a ruler. Conformation was assessed based on a scale of 1–5 (1, very flat; 2, flat; 3, medium; 4, round; 5, very round).

### 2.8. Meat Quality Traits

The color of the meat (L* = meat lightness; a* = meat redness; and b* = meat yellowness) was measured on the longissimus thoracis et lumborum (LTL) muscle at 24 h post-mortem using a Minolta color-guide (BYK-Gardner GmbH, Geretsried, Germany). The water-holding capacity (WHC) was determined as the amount of water expressed from fresh meat held under pressure (60 kg pressure) using the filter-paper press. For drip loss determination, 30 g of LTL samples were suspended in a sealed plastic sample bottles under atmospheric pressure at a temperature of 4 °C for 72 h. Samples were then gently dried with paper towels and reweighed. Drip loss (%) was calculated as the ratio of weight loss to initial sample weight. Cooking loss (%) was determined on pre-weighed, surface fat-free LTL samples that were heated in a forced-air oven at 90 °C for 45 min. Cooked samples were cooled, dried with a paper towel and reweighed. Cooking loss (%) was calculated as the proportion of weight loss upon cooking to the initial sample weight. Cooked meat samples were subsequently sheared using a Warner-Bratzler shear device mounted on a texture analyzer (TA.XT plus, Stable Micro Systems, Surrey, UK) to determine meat tenderness measured as shear force (Newtons).

### 2.9. Statistical Analysis

Data on growth performance, hematology and meat quality were analyzed using the general linear model procedure of SAS [23], according to the following statistical linear model:

*Y_ij_* = *μ* + *D_i_* + *E_ij_* (2)

where *Y_ij_* denotes response variables (growth parameters, hematology parameters, serum biochemistry and meat quality parameters), *μ* is the overall mean, *D_i_* is the effect of dietary treatment, and *E_ij_* is the random error term. Means were separated using the Tukey’s procedure. Statistical significance was declared at *p* ≤ 0.05.

## 3. Results

### 3.1. Feed Intake and Growth Performance

The effect of dietary treatments on feed intake and growth performance of lambs is shown in Table 3. There was no dietary effect (*p* > 0.05) on feed intake, average daily gain and feed conversion ratio (FCR) of the lambs. The FCR of the lambs ranged between 5.42 and 5.89.

### 3.2. Blood Parameters

The effect of partial or complete replacement of SBM with untreated or heat-treated CM on the hematological parameters of finishing lambs is presented in Table 4. Dietary treatments had no effect (*p* > 0.05) on all the hematological parameters, except for hemoglobin (Hgb) content. Higher concentration of Hgb (13.34 g/dL) was observed for sheep reared on the control diet. The concentration of Hgb was similar in CM50, CM100, HCM50 and HCM100 sheep, but was lower than that observed in CON sheep.

The effect of partial or complete substitution of dietary SBM with untreated or heated canola meal on the serum biochemistry of finishing lambs is presented in Table 5. Dietary treatments had a significant (*p* < 0.05) effect on the serum concentration of aspartate aminotransferase (AST). The highest AST concentration (159.88 IU/L) was observed in sheep reared on CM100 diet, while the lowest concentration (103.25 IU/L) was observed in sheep reared on HCM100. Total protein, albumin and creatinine concentrations in the serum of lambs were not affected (*p* < 0.05) by dietary treatments.

### 3.3. Carcass and Meat Quality Parameters

Dietary treatments had no effect on slaughter weight, fat score, HCW, CCW, dressing percentage and carcass length (Table 6). All carcasses were classified into the A grade and had a body conformation of 3. The carcasses’ fat score ranged between 3.75 and 4.67.

Dietary treatment had no effect (*p* > 0.05) on all measured meat quality parameters (Table 7). The parameters were in the range of 7.69–9.27 N for shear force, 27.6–30.5% for cooking loss, 12.8–14.8% for drip loss, 5.53–5.85 for meat pH and 4.28–5.01% for water-holding capacity.

## 4. Discussion

### 4.1. Feed Intake and Growth Performance

The observed similar feed intake among treatment groups in the present study could be because the experimental diets were isocaloric. In rapidly growing ruminants that are offered adequate protein, feed intake is mostly regulated by dietary energy concentration [24]. Therefore, optimal energy provision in the diets meant that heat treatment of the protein source would have no effect on feed intake. This could also be the reason for the absence of differences in FCR and ADG across experimental groups in this study. Several scholars have reported similar findings when groundnut, sunflower and cottonseed meals were used as dietary protein sources in the place of SBM [25,26,27]. Nevertheless, a positive effect of CM inclusion on feed intake and FCR of lambs was reported by Wiese et al. [28] and Khalid et al. [29]. The lack of effect of heat-treated CM in diets on ADG of lambs regardless of substitution level is in line with findings from other studies [30,31,32] that reported no difference in weight gain of lambs when protein oilcakes were used to replace SBM. However, the lack of improvement in weight gain when lambs were fed heated CM compared to raw CM was rather surprising given that heat treatment was expected to increase rumen bypass protein and thus improve animal performance. Indeed, higher fiber fractions (NDF, ADF and ADL) observed in diets containing heat-treated CM (Table 2) suggested the presence of insoluble Maillard products that should have increased rumen bypass protein. The heat treatment of CM was expected to improve the bypass value of CM, thereby increasing the supply of protein available for digestion in the small intestine and improving the weight gain of the lambs as observed in other studies [33]. However, post-rumen digestibility of Maillard products is not guaranteed, resulting in no beneficial heat-treatment effects, at best, or reduced protein supply, at worst. The general lack of differences in growth performance across all diets support the hypothesis that CM-containing diets promote similar growth performance as the SBM-based commercial lamb fattening diet.

### 4.2. Hematology and Serum Biochemistry

Hematological parameters are used as indicators of both the health and nutritional status of animals. The MCV for lambs in this study was within the normal range, while MCH values were higher than the normal range [34]. Blood parameters such as MCV, MCH, and MCHC are valuable for monitoring feed toxicity, especially with feed ingredients such as canola meal that may contain anti-nutritional compounds. Indeed, CM has been reported to contain anti-nutritional factors such as glucosinolates, tannins, phytic acid and sinapine that potentially exert negative effects on the growth performance and health of the animal [35,36]. Hemoglobin concentrations in this investigation were similar to those obtained by Njidda et al. [37] in different sheep breeds. Even though lambs on CM-containing diets had lower Hgb content, these values fell within the normal reference range (9.0–15 g/dL) as reported in the Merck Veterinary Manual [38]. In general, low Hgb concentration is an indication of poor nutrition [39], but this did not negatively affect the growth performance or meat quality in lambs fattened with CM-containing diets. Albumin is a sarcoplasmic protein that is responsible for protein metabolism in animal cells. Serum ALB has a close relationship with efficiency measures like dry matter intake and nutrient supply [40]. The lack of differences in serum total protein and albumin was because lambs in different treatment groups consumed similar quantities of the isocaloric and isonitrogenous diets. It was expected that lambs reared on heat-treated CM would show higher levels of serum protein as an indication of greater levels of absorbed essential amino acids from the post-rumen, facilitated by greater amounts of bypass protein. The serum activity of AST in this study fell within the normal reference range for sheep (60–280 IU/L) [41]. Aspartate aminotransferase is an enzyme that is found in the heart and liver muscle and it plays an important role in the metabolism of amino acids [42]. The findings of this study, therefore, indicated that none of the CM-containing dietary treatments contained anti-nutritional compounds with cytotoxic properties. Indeed, Paracova et al. [43] reported that damage to cell membranes results in high concentration of AST in the blood. In ruminants, protein metabolism can also be evaluated by serum creatinine levels because this biochemical is positively correlated with muscle mass and negatively correlated with backfat thickness [44].

### 4.3. Carcass and Meat Quality Traits

The observed similar slaughter weights, HCW, CCW and killing-out percentage of the lambs across treatment groups in this study corroborated the findings of Sekali et al. [45] in Mutton Merino lambs. This could be because all lambs had similar weight gain and final weights. Generally, animals with higher weight gains tend to be heavy at slaughters and have heavier carcasses [46]. The acceptable ultimate pH_u_ value for lamb meat as reported by Majdoub-Mathlouthi et al. [47] ranges from 5.6 to 6.4. Therefore, the pH value for lambs in all treatments were within the expected range irrespective of the diet. Similar observations were made in Mutton Merino lambs [45] and cross lambs (Merino/Border Leicester ewes × Poll Dorset ram) [48].

The shear force values obtained in the current study were lower than those reported by Starkey et al. [49]. A curvilinear relationship often exists between tenderness and pH [50]. Lamb meat in this study was tender since the meat pH_u_ (5.53–5.85) was lower than this range. Shear force measures the amount of force needed to cut through a piece of meat and values of less than 5 kg/cm^2^ are indicative of tender meat [51]. Color is a major element of meat quality that influences consumers’ purchase decisions [52]. Meat lightness (L*) and redness (a*) tend to increase with increase in weight and age at slaughter [53]. The redness (a*) values found in this study were, however, within the threshold value of ≥9.50 for lambs as reported by Chikwanha et al. [54]. The water-holding capacity of meat is described as the ability of meat to retain water [55]. It is an essential quality parameter that determines the visual acceptability of meat and affects the amount of water loss during transportation, storage, processing and cooking [56]. Water losses during cooking comes from juices expelled due to protein denaturation and muscle shrinkage [57]. The lack of difference in WHC, cooking loss and drip loss in this study showed that reducing or excluding SBM and using untreated or heat-treated CM in its place did not compromise meat quality in finishing Meatmaster lambs.

## 5. Conclusions

Findings from the current study showed that heat treatment of CM had no effect on growth, physiological and meat quality parameters. However, the results confirmed that CM can be included in lamb diets without compromising their nutritional and health status, growth performance and meat quality. It was concluded that heat treatment of canola meal is not an effective strategy to improve the protein value of canola meal for the growth performance and meat quality of Meatmaster lambs.

## Figures and Tables

**Table 1:** Gross composition (g/kg) of the canola meal-containing experimental diets.

Ingredients	Experimental Diets ^1^				
	CON	CM50	CM100	HCM50	HCM100
Coarse yellow maize	383.00	383.00	383.00	383.00	383.00
Soybean meal	253.00	126.50	0.00	126.50	0.00
Lucerne hay	122.00	122.00	122.00	122.00	122.00
Wheat bran	90.00	80.00	70.00	80.00	70.00
Soyhulls	79.00	69.50	60.00	69.50	60.00
Sugarcane molasses	43.00	43.00	43.00	43.00	43.00
Limestone powder	15.00	15.00	15.00	15.00	15.00
Salt coarse	6.00	6.00	6.00	6.00	6.00
Ammonium chloride	6.00	6.00	6.00	6.00	6.00
Premix B	2.20	2.20	2.20	2.20	2.20
Urea	0.80	0.80	0.80	0.80	0.80
Untreated canola meal	0.00	146.09	292.20	0.00	0.00
Heated canola meal	0.00	0.00	0.00	146.09	292.20

^1^ Experimental diets: CON, commercial lamb finishing diet in which soybean meal (SBM) was the major protein source; CM50, commercial finishing diet in which 50% of SBM was replaced with untreated canola meal; CM100, commercial finishing diet in which all SBM was replaced with untreated canola meal; HCM50, commercial finishing diet in which 50% of SBM was replaced with heat-treated canola meal; and HCM100, commercial finishing diet in which all SBM was replaced with heat-treated canola meal.

**Table 2:** Chemical composition of experimental diets (g/kg dry matter (DM), unless otherwise stated).

Parameters	Experimental Diets ^1^				
	CON	CM50	CM100	HCM50	HCM100
Dry matter (g/kg)	894.99	894.42	893.93	905.68	900.35
Ash	86.53	68.38	68.39	65.15	72.99
Organic matter	808.45	826.04	825.54	840.53	827.36
Crude protein	160.1	160.2	160.0	160.1	160.1
Neutral detergent fiber	156.23	195.51	220.62	307.41	307.32
Acid detergent fiber	103.65	116.81	122.58	146.17	126.29
Acid detergent lignin	30.35	53.62	53.94	63.08	64.08

^1^ Experimental diets: CON, commercial lamb finishing diet in which SBM was the major protein source; CM50, commercial finishing diet in which 50% of SBM was replaced with untreated canola meal; CM100, commercial finishing diet in which all SBM was replaced with untreated canola meal; HCM50, commercial finishing diet in which 50% of SBM was replaced with heat-treated canola meal; and HCM100, commercial finishing diet in which all SBM was replaced with heat-treated canola meal.

**Table 3:** Dietary effect of partially or completely replacing soybean meal with untreated or heat-treated canola meal on feed intake and growth performance of Meatmaster lambs.

Parameters ^1^	Experimental Diets ^2^					SEM ^3^	*p*-Value
	Control	CM50	CM100	HCM50	HCM100		
Initial weight (kg)	24.2	24.6	24.7	25.4	24.9	1.31	0.978
ADFI (kg/day)	1.26	1.24	1.26	1.25	1.21	0.05	0.931
ADG (kg/day)	0.23	0.22	0.22	0.23	0.21	0.01	0.180
FCR	5.47	5.81	5.73	5.42	5.89	0.21	0.401

^1^ Parameters: ADFI, average daily feed intake; ADG, average daily gain; FCR, feed conversion ratio. ^2^ Experimental diets: CON, commercial lamb finishing diet in which SBM was the major protein source; CM50, commercial finishing diet in which 50% of SBM was replaced with untreated canola meal; CM100, commercial finishing diet in which all SBM was replaced with untreated canola meal; HCM50, commercial finishing diet in which 50% of SBM was replaced with heat-treated canola meal; and HCM100, commercial finishing diet in which all SBM was replaced with heat-treated canola meal. ^3^ SEM, standard error mean.

**Table 4:** Effect of partial or complete replacement of dietary soybean meal with untreated or heat-treated canola meal on hematological characteristics of Meatmaster lambs.

Parameters ^1^	Experimental Diets ^2^					SEM ^3^	*p*-Value
	CON	CM50	CM100	HCM50	HCM100		
Erythrocytes (×10^12^/L)	8.72	8.70	8.90	6.74	8.04	0.54	0.104
Hemoglobin (g/dL)	13.34 ^a^	12.26 ^ab^	12.58 ^ab^	11.75^b^	11.59 ^b^	0.33	0.006
Hematocrit (L/L)	0.29	0.29	0.29	0.26	0.25	0.02	0.262
MCV (fL)	33.29	33.24	34.07	31.63	31.48	1.26	0.502
MCH (pg)	15.43	14.23	15.88	14.60	14.48	0.66	0.440
MCHC (g/dL)	46.24	43.60	46.53	40.39	46.00	2.75	0.325
RDW	11.50	11.49	11.91	29.86	8.94	7.61	0.426
Leucocytes (×10^9^/L)	10.41	8.43	8.09	7.61	10.14	0.90	0.167
Neutrophils (×10^9^/L)	3.14	2.33	3.63	3.30	3.13	0.57	0.635
Lymphocytes (×10^9^/L)	6.83	5.60	4.00	4.95	6.59	0.74	0.100
Monocytes (×10^9^/L)	0.39	0.39	0.33	0.19	0.30	0.07	0.363
Eosinophils (×10^9^/L)	0.05	0.11	0.13	0.10	0.12	0.04	0.751
Platelets (×10^9^/L)	281.0	310.9	327.4	226.4	343.6	37.37	0.329

^ab^ Means within a row that do not share a common superscript differ significantly (*p* < 0.05). ^1^ Parameters: MCV, mean corpuscular volume; MCH, mean corpuscular hemoglobin; MCHC, mean corpuscular hemoglobin concentration; RDW, red cell distribution width. ^3^ SEM, standard error of the mean. ^2^ Experimental diets: CON, commercial lamb finishing diet in which SBM was the major protein source; CM50, commercial finishing diet in which 50% of SBM was replaced with untreated canola meal; CM100, commercial finishing diet in which all SBM was replaced with untreated canola meal; HCM50, commercial finishing diet in which 50% of SBM was replaced with heat-treated canola meal; and HCM100, commercial finishing diet in which all SBM was replaced with heat-treated canola meal.

**Table 5:** Serum biochemical parameters of Meatmaster lambs that were fed untreated or heat-treated canola meal as a partial or complete replacement for soybean meal in finishing diets.

Parameters ^1^	Experimental Diets ^2^					SEM ^3^	*p*-Value
	CON	CM50	CM100	HCM50	HCM100		
Total protein (g/L)	69.63	72.00	71.00	68.71	70.63	1.19	0.390
Albumin (g/L)	40.25	40.88	40.50	39.71	40.13	0.69	0.827
AST (IU/L)	133.1 ^ab^	135.7 ^ab^	159.88 ^a^	129.14 ^ab^	103.25 ^b^	13.48	0.036
Creatinine (µmol/L)	53.25	61.25	58.75	56.57	64.13	3.53	0.256

^ab^ Means within a row that do not share a common superscript differ significantly (*p* < 0.05). ^1^ Parameters: AST, aspartate transaminase. ^2^ Experimental diets: CON, commercial lamb finishing diet in which SBM was the major protein source; CM50, commercial finishing diet in which 50% of SBM was replaced with untreated canola meal; CM100, commercial finishing diet in which all SBM was replaced with untreated canola meal; HCM50, commercial finishing diet in which 50% of SBM was replaced with heat-treated canola meal; and HCM100, commercial finishing diet in which all SBM was replaced with heat-treated canola meal. ^3^ SEM, standard error of the mean.

**Table 6:** Effect of partial or complete replacement of dietary soybean meal with untreated or heat-treated canola meal on carcass traits of Meatmaster lambs.

Carcass Traits	Experimental Diets ^1^					SEM ^2^	*p*-Value
	CON	CM50	CM100	HCM50	HCM100		
Slaughter weight (kg)	42.0	41.2	41.6	43.3	40.7	1.62	0.850
Fat score	4.25	4.25	4.25	4.67	3.75	0.45	0.763
Hot carcass weight (kg)	20.4	20.4	20.2	20.3	19.6	0.90	0.965
Cold carcass weight (kg)	20.0	20.0	19.8	19.9	19.2	0.89	0.963
Carcass length (cm)	63.9	61.5	64.0	65.0	64.8	1.03	0.166
Dressing %	48.5	49.3	48.6	46.4	48.0	0.73	0.150

^1^ Experimental diets: CON, commercial lamb finishing diet in which SBM was the major protein source; CM50, commercial finishing diet in which 50% of SBM was replaced with untreated canola meal; CM100, commercial finishing diet in which all SBM was replaced with untreated canola meal; HCM50, commercial finishing diet in which 50% of SBM was replaced with heat-treated canola meal; and HCM100, commercial finishing diet in which all SBM was replaced with heat-treated canola meal. ^2^ SEM, standard error of the mean.

**Table 7:** Meat quality parameters of Meatmaster lambs that were fed untreated or heat-treated canola meal as a partial or complete replacement for soybean meal in finishing diets.

Meat Quality Parameters	Experimental Diets ^1^					SEM ^2^	*p*-Value
	CON	CM50	CM100	HCM50	HCM100		
pH_u_	5.85	5.56	5.73	5.69	5.53	0.10	0.219
Shear force (N)	8.08	7.69	8.29	9.27	7.88	0.74	0.689
Cooking loss (%)	29.5	28.4	30.5	29.7	27.6	0.59	0.517
Drip loss (%)	12.8	13.1	13.0	14.8	13.5	0.86	0.598
Water-holding capacity (%)	5.01	4.30	4.28	4.63	5.57	1.25	0.512
Meat lightness (L*)	37.8	39.2	39.8	41.8	39.5	1.29	0.383
Meat redness (a*)	13.4	13.1	12.7	11.7	13.4	1.05	0.822
Meat yellowness (b*)	13.5	14.1	13.6	14.3	15.3	0.89	0.673

^1^ Experimental diets: CON, commercial lamb finishing diet in which SBM was the major protein source; CM50, commercial finishing diet in which 50% of SBM was replaced with untreated canola meal; CM100, commercial finishing diet in which all SBM was replaced with untreated canola meal; HCM50, commercial finishing diet in which 50% of SBM was replaced with heat-treated canola meal; and HCM100, commercial finishing diet in which all SBM was replaced with heat-treated canola meal. ^2^ SEM, standard error of the mean.
